# PEGylated liposome IHL-305 markedly improved the survival of ovarian cancer peritoneal metastasis in mouse

**DOI:** 10.1186/1471-2407-12-462

**Published:** 2012-10-10

**Authors:** Hiroaki Konishi, Akimitsu Takagi, Akinobu Kurita, Norimasa Kaneda, Takeshi Matsuzaki

**Affiliations:** 1 Yakult Central Institute for Microbiological Research, 1790 Yaho, Kunitachi, Tokyo, 1860-8650, Japan

**Keywords:** Ovarian cancer, Peritoneal metastasis, Ascites fluid, Spheroid culture, Drug delivery system

## Abstract

**Background:**

Advanced ovarian cancer is characterized by peritoneal metastasis and the accumulation of ascites. Peritoneal metastasis of ovarian cancer is a major cause of the negative treatment outcome, as these metastases are resistant to most chemotherapy regimens. The aim of this study was to clarify aggressive pathology of peritoneal metastasis and examine the therapeutic efficacy of a liposomal agent in the model.

**Methods:**

A human cancer cell line ES-2 of ovarian clear cell carcinoma, known as a chemotherapy-resistant cancer, was cultured in nonadherent plate to form spheroid and single cell suspension was transplanted into mouse peritoneal cavity. The epidermal growth factor receptor (EGFR) pathways in the cellular aggregates were analyzed both spheroid and ascites. The pharmacokinetics and therapeutic efficacy of CPT-11 (45 mg/kg) and IHL-305 (45 mg/kg), an irinotecan-encapsulated liposome, were examined by intravenous administration.

**Results:**

Established peritoneal metastasis model showed an accumulation of ascites. The activation of EGFR and Akt was demonstrated in cellular aggregates both in the spheroid and ascites. In ascites samples, the area under the curve of SN-38, the activated form of CPT-11, was 3.8 times higher from IHL-305-treated mice than from CPT-11-treated mice. IHL-305 prolonged the survival time and decreased the accumulation of ascites and tumor metastasis. The median survival time were 22, 37 and 54 days in the control, CPT-11-treated, and IHL-305-treated mice, respectively.

**Conclusions:**

EGFR/Akt pathway contributes to the aggressive progression in ES-2 peritoneal metastasis model and effective delivery into ascites of IHL-305 was thought to useful treatment for ovarian cancer with peritoneal metastasis.

## Background

About 14,000 people die from ovarian cancer every year in the United States [1]. The five-year survival rate of patients with ovarian cancer is lower than that for other gynecologic cancers. Unlike other cancers, ovarian cancer cells spread widely throughout the peritoneal cavity because of the absence of an anatomical barrier. The relatively poor prognosis for patients with ovarian cancer is due to a frequently advanced disease stage with peritoneal metastasis at the time of diagnosis [2], and a substantially lower survival rate for advanced stages than for early stages [3]. To improve the survival rate of patients with ovarian cancer, many efforts have been made to detect ovarian cancer at an early stage before peritoneal metastasis had occurred.

CA-125 is used as a tumor marker to detect ovarian cancer, but it is not a reliable marker for early-stage disease [4]. In addition, the subjective symptoms are imprecise [2]. Currently, the detection of early-stage ovarian cancer is extremely difficult. For the treatment of ovarian cancer with peritoneal metastasis, the intraperitoneal administration of cisplatin or paclitaxel has been examined in clinical trials. Though some of these trials were successful [3,5-7], other trials did not succeed because of chemical peritonitis and the enhancement of adverse effects [8]. On the other hand, intravenous administration has been shown to have a minimal effect [9]. None of the intraperitoneal or intravenous chemotherapy regimens have been confirmed as effective against ovarian cancer with peritoneal metastasis. Thus, a novel therapy for the treatment of ovarian cancer with peritoneal metastasis is needed.

Some experimental studies have been conducted to characterize the mechanism of peritoneal metastasis. Heparin-binding EGF-like growth factor (HB-EGF), stromal cell-derived factor 1alpha (SDF-1α)/CXC receptor 4 and vascular cell adhesion molecule-1 (VCAM-1) have been reported to be related to the progression of peritoneal metastasis in *ex vivo* studies [10-12], and the activation of HER2, a member of the epidermal growth factor receptor (EGFR) family, has been reported in spheroid cultures [13]. The activation of EGFR pathways has been shown to be involved in tumorigenesis in many cancers [14]. Spheroid cultures are regarded as a suitable *in vitro* model of peritoneal metastasis especially with regard to their anchorage-independent growth, which resembles floating cancer cell aggregates in ascites; however, the EGFR pathways in spheroid cultures are not fully understood.

In peritoneal metastasis, it is difficult to resect cancer cells completely because the cells spread throughout the peritoneal cavity. Consequently, the delivery and maintenance of anti-cancer agents in the peritoneal cavity is an important therapeutic strategy. Liposome delivery has been demonstrated to improve the pharmacokinetic profile and therapeutic efficacy of various anti-cancer agents [15,16]. Improved efficacy is in part a result of the passive targeting of tumor sites based on the enhanced permeability and retention (EPR) effect. Liposomes are captured by the reticuloendothelial system (RES); however, liposomes with surfaces that have been modified with polyethylene glycol (PEG) are able to avoid uptake by the RES and have a longer retention time in the blood [17]. Irinotecan hydrochloride (CPT-11), which acts by inhibiting DNA topoisomerase I, is widely used against colorectal and ovarian cancer because of its robust efficacy [18,19]. IHL-305 has developed as an irinotecan-encapsulated and PEGylated liposome, and its antitumor-activity have been demonstrated in mouse model [20]. Efficient delivery of PEGylated liposome encapsulated with vinorelbine and doxorubicin into ascites fluid have been reported [21,22]. Thus, we hypothesized that liposomal formulations of anti-cancer agents might be useful for the treatment of peritoneal metastasis.

In this study, a murine model for the peritoneal metastasis of ovarian cancer was established using a human clear cancer cell line. This model was accompanied by accumulation of ascites, and EGFR pathways were activated in cellular aggregates collected from the ascites. Intravenously administrated IHL-305 was efficiently delivered into peritoneal ascites; this drug delivery system prolonged the survival of the mouse with peritoneal metastasis.

## Results

### Establishment of ES-2 peritoneal metastasis mouse model

**Figure 1:**
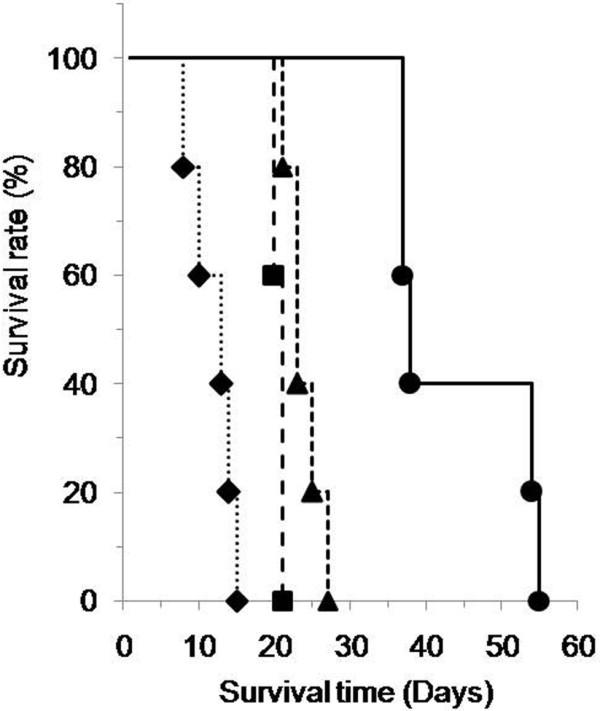
**Relation between the number of inoculated ES-2 cells and the survival of the inoculated mice.** Various numbers of ES-2 human ovarian cancer cells (2 × 10^4^ cells (●), 2 × 10^5^ cells (▲), 2 × 10^6^ cells (■), 2 × 10^7^ cells (♦)) were inoculated into the peritoneal cavity of mice. After inoculation, the survival time of the mice was examined (n=5, each group).

Various numbers of ES-2 human ovarian cancer cells were inoculated into the peritoneal cavities of mice, and the survival and performance statuses after inoculation were observed. The survival time decreased depending on the number of inoculated cells (Figure 1). The accumulation of ascites and the formation of metastatic tumor nodules surrounding organs in the peritoneal cavity were observed after the inoculation of 2 × 10^4^ to 10^6^ ES-2 cells. Floating cell aggregates were also observed in the ascites. Mice inoculated with 2 × 10^7^ ES-2 cells had the shortest survival period and did not exhibit the accumulation of ascites. The optimal number of inoculated cells was decided to be 2 × 10^5^ cells because mice inoculated with this number of cells exhibited characteristics of peritoneal metastasis such as the accumulation of ascites and the formation of metastases.

### EGFR pathways in ES-2 cells of spheroid cultures *in vitro* and in ascites *in vivo*

**Figure 2:**
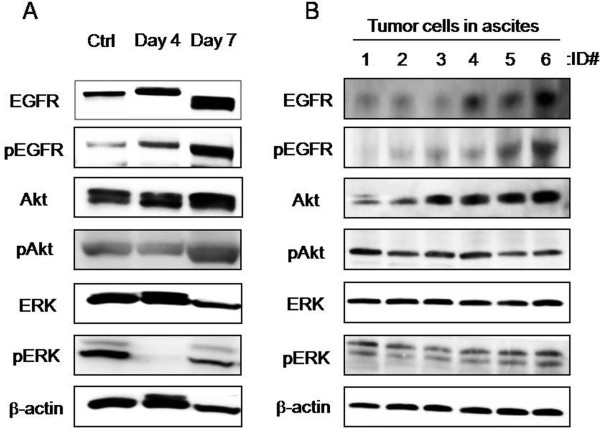
**Activation of EGFR pathways in both *in vitro *and *in vivo *models of peritoneal metastasis.** The statuses of EGFR signaling molecules (EGFR, Akt and ERK) were examined in ES-2 cells in spheroid cultures and in ascites using an immunoblotting analysis. Phosphorylation status was examined to evaluate the activated forms of these proteins. The analyzed sites of phosphorylation on EGFR, Akt and ERK were Tyr^1069^, Ser^473^ and Thr^202^/Tyr^204^, respectively. (**A**) ES-2 cells were seeded into a spheroid culture plate on day 1 and were analyzed on days 4 and 7. Ctrl: monolayer culture. (**B**) ES-2 cell aggregates in ascites were collected from mice with peritoneal metastasis (n=6, mouse ID#1-6)

Tumor cell aggregates that were detached and floating in ascites were observed in the mouse models. When attached cells lose their anchorage, a form of cell death known as anoikis is induced. Spheroids are regarded as an anchorage-independent growth experimental model, and the involvement of Akt has been reported in anoikis resistance [23]. In ES-2 spheroids, the phosphorylation of Akt was detected, and the phosphorylation of EGFR and ERK1/2, an EGFR downstream molecule involved in a pathway other than the one involving Akt, also detected (Figure 2A). However, the phosphorylation of ERK1/2 was transiently down-regulated in the process of growth. Likewise, the phosphorylation of both EGFR and Akt and ERK1/2 were observed in cancer cell aggregates in ascites (Figure 2B). Thus, EGFR and its downstream signals were clearly up-regulated in the ES-2 cancer cell-induced peritoneal metastasis models both *in vitro* and *in vivo*.

### Cytotoxic activity of anti-cancer agents against ES-2 monolayer and spheroids

**Figure 3:**
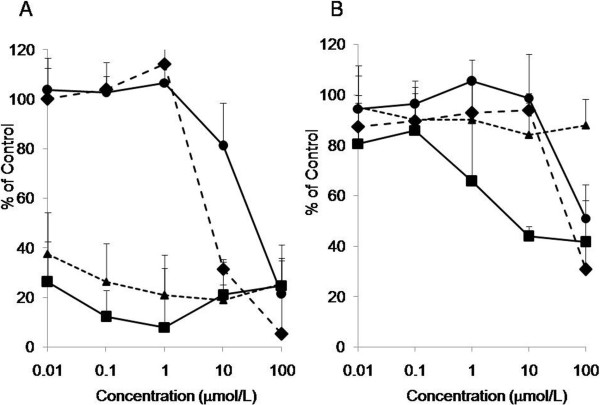
**Cytotoxicity of anti-cancer agents against ES-2 cell monolayers and spheroids.** ES-2 cell monolayers (**A**) and spheroids (**B**) were exposed to cisplatin (●), paclitaxel (▲), SN-38 (■) and PD153035 (♦) for 48 hr. The cytotoxic activity was measured using an MTS assay (n=3 per group calculated from 3 independent experiments). The percentage of viable cells is shown relative to untreated controls.

The cytotoxic activity of anti-cancer agents against ES-2 monolayer and spheroids, an *in vitro* model of peritoneal metastasis, was compared using cisplatin (a platinum drug, CDDP), paclitaxel (a tubulin inhibitor, PTX), SN-38 (a topoisomerase I inhibitor, an active metabolite of CPT-11), and PD153035 (an EGFR inhibitor). CDDP, PTX and CPT-11 are clinically used against ovarian cancers [24]. In general, the activities of the anti-cancer agents were reduced in the spheroids [25]. The IC_50_ values of CDDP, PTX, SN-38 and PD153035 in monolayer were 5.2 μM, <0.01 μM, <0.01 μM, 6.0 μM, respectively (Figure 3A). The IC_50_ values of CDDP and PTX in spheroids could not be estimated because of their weak activities (Figure 3B). On the other hand, the IC_50_ values of SN-38 and PD153035 in spheroids were 5.2 μM and 50 μM, respectively. Among the four agents that were assayed, SN-38 exerted a relatively strong cytotoxicity.

### Pharmacokinetic profiles of CPT-11 and IHL-305

**Figure 4:**
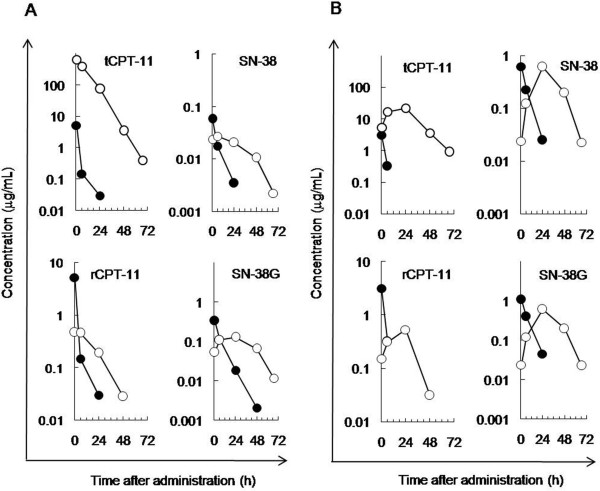
**Pharmacokinetic profile of IHL-305 in an ES-2 peritoneal metastasis mouse model.** CPT-11 (45 mg/kg: ●) or IHL-305 (45 mg/kg: ○) was intravenously administered at 0 time to mice with peritoneal metastasis. The concentrations of total CPT-11 (tCPT-11), released CPT-11 from the liposome (rCPT-11), SN-38 and SN-38 glucuronide (SN-38G) were determined in (**A**) blood and (**B**) ascites.

The metabolic conversion from CPT-11 to SN-38 by esterase is necessary for the anti-cancer activity of CPT-11. For the treatment of peritoneal metastasis, the delivery of anti-cancer agents to detached and floating cancer cells in ascites is vital for therapy. The pharmacokinetic profiles of CPT-11 and its liposomal formulation, IHL-305, were examined in blood and ascites samples collected from mice with peritoneal metastasis to determine whether CPT-11, once released from IHL-305, is delivered to the ascites and converted to SN-38 and then to SN-38 glucuronide (SN-38G) within the ascites. For this purpose, the total amount of CPT-11 (tCPT-11), the amount of CPT-11 that is released from IHL-305 (rCPT-11), and the amounts of SN-38 and SN-38G were determined using HPLC. The results indicated that the pharmacokinetic profile of CPT-11 and its metabolites were improved by the liposomal formulation. In the blood samples, the concentrations of tCPT-11, rCPT-11, SN-38 and SN-38G rapidly decreased after administration in CPT-11-treated mice. In the IHL-305-treated mice, however, the concentrations decreased slowly after administration (Figure 4A). The AUC of SN-38 in the IHL-305-treated mice was 2.8 times higher than that in the CPT-11-treated mice. In ascites samples, the concentrations of tCPT-11, rCPT-11, SN-38 and SN-38G rapidly decreased just after administration in the CPT-11-treated mice. In the IHL-305-treated mice, however, the concentrations reached a maximum at 24 hr after administration and then slowly decreased (Figure 4B). The AUC of SN-38 in the IHL-305-treated mice was 3.8 times higher than that in the CPT-11-treated mice.

### Metastatic behaviors of peritoneal metastasis in mice treated with CPT-11 or IHL-305

The typical metastatic behaviors observed in mice with peritoneal metastasis are the accumulation of ascites and metastasis to intra-abdominal organs, such as the omentum and mesenterium. As surrogate endpoints of the anti-cancer activity of CPT-11 and IHL-305, ascites and metastasis parameters were examined after the administration of these agents. CPT-11 showed an anti-cancer activity, and IHL-305 showed an even greater activity. Control mice exhibited high incidences of the accumulation of ascites and the formation of metastases on days 18 and 26, and all the mice had died by day 31 after cancer cell inoculation. The CPT-11-treated mice exhibited no accumulation of ascites on days 18 and 26, but high incidences of metastasis were observed on days 18, 26 and 31. The IHL-305-treated mice exhibited no ascites or metastasis on days 18, 26 and 31 except for one mouse who exhibited a metastatic tumor on day 18 (Table 1). The anti-cancer potency of IHL-305 was thus revealed by the strong suppression of the accumulation of ascites and metastasis.

**Table 1:** Abnormal ascites and tumor metastasis in peritoneal metastasis mice

	**Group**	**Ascites**		**Metastasis (incidence)**			
		**Incidence**	**Volume (mL)**	**Omentum**	**Diaphragm**	**Mesenterium**	**Pancreas**
	Control	7/7	2.63±0.75	3/7	3/7	4/7	4/7
Day 18	CPT-11	0/5^*^	-	3/5	0/5	4/5	0/5
	IHL-305	0/5^*^	-	1/5	0/5	1/5	0/5
	Control	2/3	2.95±0.92	3/3	3/3	3/3	2/3
Day 26	CPT-11	0/4^**^	-	3/4	3/4	4/4	1/4
	IHL-305	0/5^*^	-	0/5	0/5	0/5	0/5
	Control	-	-	-	-	-	-
Day 31	CPT-11	3/4	0.68±0.89	4/4	3/4	4/4	2/4
	IHL-305	0/5^***^	-	0/5	0/5	0/5	0/5

* p<0.01 vs. Ctrl.

** p<0.05 vs. Ctrl.

*** p<0.05 vs. CPT-11.

Analyzed by Kaplan-Meier method.

### Survival of mice with peritoneal metastasis treated with CPT-11, IHL-305

**Figure 5:**
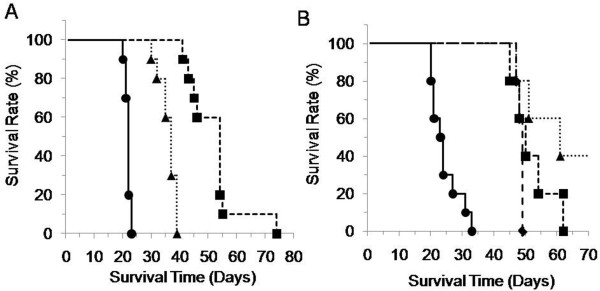
**Survival time of the ES-2 peritoneal metastasis mouse model after treatment with CPT-11 or IHL-305.** ES-2 cells (2 x 10^5^ cells) were inoculated into the peritoneal cavity of mice on day 1. (**A**) Saline as the vehicle control (●), CPT-11 (45 mg/kg/day: ▲) or IHL-305 (45 mg/kg/day: ■) was administered on days 4, 8, and 12, and the survival time was analyzed (n=10, each group). (**B**) Saline was administered as the vehicle control (●). IHL-305 (45 mg/kg) was administered on days 4, 8 and 12 (schedule A, ▲), days 8, 12 and 16 (schedule B, ■) or days 12, 16 and 20 (schedule C, ♦), and the survival time was analyzed (n=5, each group).

We demonstrated that CPT-11 and IHL-305 suppressed the accumulation of ascites and the formation of metastases as surrogate endpoints and examined the survival time of the mice as the primary endpoint. The median survival time (MST) after the inoculation of ES-2 cells was 22, 37 and 54 days in the control, CPT-11-treated and IHL-305-treated groups, respectively (Figure 5A). Both CPT-11 and IHL-305 prolonged the survival time, compared with that in the control group (CPT-11, p<0.01; IHL-305, p<0.01). IHL-305 significantly prolonged the survival time, compared with CPT-11 (p<0.01).

### Schedule-independency of IHL-305 therapy

Finally, we examined the schedule-dependency of IHL-305 on the therapeutic effect, since the efficacy of the test compound against the aggressive progression of peritoneal metastasis depends on the administration schedule [26]. After the inoculation of ES-2 cells in mice (day 1), IHL-305 was administered to mice according to various schedules as follow: days 4, 8 and 12 (schedule A), days 8, 12 and 16 (schedule B), and days 12, 16 and 20 (schedule C). The survival time was shortened in the schedule C treatment group, but the difference was not significant. The MST was 61, 50 and 49 days for the schedule A, B and C groups, respectively (Figure 5B). Thus, the survival time of the IHL-305-treated mice was prolonged independently of the administration schedule.

## Discussion

We examined the peritoneal metastasis of ovarian cancer using both *in vitro* and *in vivo* models in this experimental study. We demonstrated that the accumulation of ascites and a reduction in survival were induced by the inoculation of the ES-2 ovarian clear cell carcinoma cell line, a known chemotherapy-resistant cancer cell type, into the peritoneal cavity and that the EGFR pathways were activated in the cancer cells in both the *in vitro* and *in vivo* models. Since ES-2 cells harbor a genetic mutation in *B-raf*, a downstream member of the EGFR pathway and a known resistance factor against anti-EGFR therapy [27], EGFR interference was thought to be an unfavorable strategy in ES-2 models. Thus, we focused on the management of ascites using a modified liposomal formulation of a chemotherapeutic agent, since doxorubicin liposomes have been applied for ovarian cancer therapy. We demonstrated that IHL-305 was retained within the ascites of the mice and prolonged the survival period of the mice.

Ovarian cancers advance to peritoneal metastasis through the dissemination of the cancer cells from the ovary to the peritoneal cavity. In this study, we used a clear cell type ovarian cancer cell line, ES-2 to establish peritoneal metastasis models (Figure 1). We also examined an adenocarcinoma ovarian cell line**,** SK-OV-3, and the amount of vascular endothelial growth factor (VEGF) in the spheroid culture supernatant was much higher for the ES-2 cells than for the SK-OV-3 cells (data not shown). VEGF reportedly plays an important role in the progression of peritoneal metastasis [28]. In clinical studies, clear cell type ovarian cancer has exhibited a resistance to chemotherapy [29]. Therefore, we selected ES-2 cells as an aggressive peritoneal metastasis model in this study.

In general, peritoneal metastasis induced the accumulation of ascites, including floating cancer cell aggregates. We focused on the floating cells, which showed anchorage-independent growth and were regarded as comparable to the spheroids of cancer cells in the *in vitro* peritoneal metastasis model. We detected the activation of Akt in both the *in vivo* and the *in vitro* models of peritoneal metastasis (Figure 2). Akt reportedly plays an important role in resistance to anoikis (detachment-induced apoptosis) [23]. In addition, the activation of EGFR was also detected in both models. However, EGF, an activating ligand of EGFR, was not detected in the spheroid culture medium or ascites (data not shown). A three-dimensional culture resulting in the up-regulation of HER2 has been reported [13]. Therefore, alterations in the formation of three-dimensional structures might activate the EGFR pathways.

Platinum and taxane agents are used as standard chemotherapy for the treatment of ovarian cancers. We examined the cytotoxic activity of CDDP, SN-38, PTX and PD153035 against monolayer and spheroids of ES-2 cells. Spheroids of ES-2 cells demonstrated resistance to the agents tested, especially to CDDP and PTX, though these agents exerted potent activity in monolayer cultures (Figure 3A). Cancer cells bearing the *B-raf* gene mutation have been reported to exhibit resistance to EGFR inhibitors, and ES-2 cells carry this mutation [27]; therefore, we assumed that the ES-2 cells were resistant to the EGFR inhibitors. Targeting Akt is still remaining but among the tested agents, SN-38 exerted the highest cytotoxic activity (Figure 3B); thus, we applied CPT-11, a prodrug of SN-38, for the treatment of the peritoneal metastasis.

Liposomal formulations improve the pharmacokinetic profile, but the profile of IHL-305 in a peritoneal metastasis model with ascites has not been previously examined. In the present study, a larger amount of IHL-305 was retained in the ascites, compared with CPT-11 (Figure 4B). Generally, therapeutic agents in the peritoneal cavity are absorbed through vessels and lymph nodes. High molecular weight agents are mainly absorbed through lymph nodes, whereas low molecular weight agents are mainly absorbed through vessels [30]. In peritoneal metastasis, cancer cells invading the lymph nodes reduce the absorption by lymph nodes. The absorption rate of IHL-305, a high molecular weight agent, has been shown to be lower than that of CPT-11 (Figure 4B). It is suggested, therefore, that those improved pharmacokinetic profile of IHL-305 leads to an enhancement of the anti-cancer efficacy (Figure 5A). As shown in Table 1, ascites fluids have observed in CPT-11 group on day 31, whereas it has not observed on days 18 and 26. It was thought that this dosage of CPT-11 was not enough to cure the tumor cells completely, although this dosage was recommended dose of CPT-11 in mouse. Thus, the remaining tumor cells in CPT-11-treated mouse proliferated and then release of the ascites fluids was induced. Moreover, IHL-305 prolonged the survival time when administered according to any of the schedules that were examined (Figure 5B). IHL-305 was administered on day 12 after the inoculation of ES-2 cells; at this stage, advanced tumor invasion (omentum, pancreas, etc.) was apparent. Nevertheless, IHL-305 exhibited a therapeutic efficacy against the invaded tumors in addition to the cancer cell aggregates in the ascites.

## Conclusion

We demonstrated that the activation of EGFR pathways contributes to the aggressive progression observed in an established ES-2 peritoneal metastasis model, and IHL-305 effectively suppressed the progression of peritoneal metastasis and prolonged the survival period in this model. This liposomal formulation was considered as an effective delivery tool toward peritoneal cavity.

## Materials and methods

### Reagents

CPT-11 and SN-38 were obtained from Yakult Honsha, Co., Ltd. (Tokyo, Japan). IHL-305 was obtained from Terumo Corporation (Tokyo, Japan). PTX and CDDP were obtained from Sigma, Inc. (St. Louis, MO, USA). PD153035 was obtained from Merck KGaA (Darmstadt, Germany).

### Cell cultures

The ES-2 cell line, a human ovarian clear cell carcinoma, was obtained from American Type Culture Collection (Manassas, VA, USA). For the spheroid cultures, the ES-2 cells were seeded into a 96-well spheroid culture plate (Sumilon cell tight Multiwell plate; Sumitomo Bakelite Co., Ltd, Tokyo, Japan) on day 1 (2500 cells/well; 50 μL/well). Cells were collected on days 4 and 7 and used for the immunoblot analysis.

### Immunoblot analysis of EGFR pathways

The harvested spheroid culture cells or cancer cells from the peritoneal cavity were washed in ice-cold PBS and lysed in RIPA buffer (10 mM Tris–HCl [pH7.4], 0.1% nonidet-P-40, 0.1% sodium deoxycholate, 0.1% SDS, 150 mM NaCl, 1 mM EDTA, 10μg/mL aprotinin, and phosphatase inhibitor cocktail [Nacalai Tesque, Inc., Kyoto, Japan]). An immunoblot analysis was performed using the following antibodies: anti-EGFR (1005; Santa Cruz Biotechnologies, Inc., California, CA, USA), anti-Akt1/2/3 (H-136; Santa Cruz Biotechnologies), anti-ERK2 (C-14; Santa Cruz Biotechnologies), anti-β-actin (N-21; Santa Cruz Biotechnologies), anti-phospho-EGFR (Tyr^1069^; Cell Signaling Technology [CST], Inc., Beverly, MA, USA), anti-phospho-Akt(193 H12, Ser^473^; CST) and anti-phopho-p44/42 MAPK (Erk1/2) (D13.14.4E, Thr^202^/Tyr^204^; CST). All the antibodies were used at a dilution of 1:1000 except for p-MAPK and β-Actin, which were used at a dilution of 1:2000. All other chemicals were of the highest purity available.

### Cytotoxicity of anti-cancer agents against monolayers and spheroids of ES-2 cells

ES-2 cells were seeded into a 96-well plate for monolayers or spheroid culture on day 1 (2500 cells/well) and exposed to anti-cancer agents for 48 h on days 2 to 4 (monolayers) or days 5 to 7 (spheroids). The number of viable cells was determined after exposure using the CellTiter 96 Aqueous One Solution Cell Proliferation Assay (Promega Corporation, Madison, WI, USA).

### Laboratory animals

Four-week-old female BALB/c nu/nu mice were obtained from Japan SLC, Inc. (Hamamatsu, Japan). All of the *in vivo* experimental protocols were approved by the animal care committee of the Yakult Central Institute for Microbiological Research.

### Therapeutic study of CPT-11 and IHL-305 in peritoneal metastasis mouse model

The efficacy of CPT-11 and IHL-305 against peritoneal metastasis was then evaluated after the inoculation of ES-2 cells (2 × 10^5^ cells/200 μL saline) into the peritoneal cavity (day 1) and the random division of the mice into test groups. CPT-11 (45 mg/kg/day) or IHL-305 (45 mg/kg/day) was administered via the tail vein on days 4, 8 and 12. The status of peritoneal metastasis was then evaluated on days 18, 26 and 31 by monitoring the volume of ascites and tumor nodules in the omentum, diaphragm, mesenterium and pancreas. The schedule dependency of IHL-305 was also examined. IHL-305 (45 mg/kg/dose) was administered via the tail vein to the mice on days 4, 8 and 12, days 8, 12 and 16, or days 12, 16 and 20. The survival time of the mice receiving the various therapies were then compared.

### Pharmacokinetic profiles of CPT-11 and IHL-305 in mice with peritoneal metastasis

About 20 days after the inoculation of ES-2 cells (2 × 10^5^ cells) into the peritoneal cavity, CPT-11 (45 mg/kg) or IHL-305 (45 mg/kg) was administered via the tail vein to mice exhibiting the macroscopic accumulation of ascites. Under anesthesia, blood and ascites were excised at 0.5, 6, 24, 48 and 67 hours after administration. The concentration of total CPT-11 (tCPT-11), released CPT-11 from the liposome (rCPT-11), SN-38 and SN-38 glucuronide (SN-38G) were determined using high-performance liquid chromatography (HPLC).

### Statistical analysis

The incidence of abnormal ascites and the survival time were analyzed using the Kaplan-Meier method. Probability values of less than 5% were considered significant.

## Abbreviations

HB-EGF: Heparin-binding EGF-like growth factor; SDF-1α: Stromal cell-derived factor 1alpha; VCAM-1: Vascular cell adhesion molecule-1; EGFR: Epidermal growth factor receptor; EPR: Enhanced permeability and retention; RES: Reticuloendothelial system; PEG: Polyethylene glycol; CPT-11: Irinotecan hydrochloride; PTX: Paclitaxel; CDDP: Cisplatin; VEGF: Vascular endothelial growth factor.

## Competing interest

All authors are employment of Yakult Honsha Co., Ltd.

## Authors’ contributions

AT directed this study. HK and AT participated in the acquisition, analysis and interpretation of all data. AK and NK conducted pharmacokinetic analysis. TM participated in the management of IHL-305 among provider. All authors read and approved the final manuscript.

## Pre-publication history

The pre-publication history for this paper can be accessed here:

http://www.biomedcentral.com/1471-2407/12/462/prepub

## References

[B1] American Cancer Society Cancer Facts & Figures 2010 2010 Atlanta: American Cancer Society

[B2] Rossing MA Wicklund KG Cushing-Haugen KL Weiss NS Predictive value of symptoms for early detection of ovarian cancer J Natl Cancer Inst 2010 102 222 229 10.1093/jnci/djp500 20110551 PMC2826180

[B3] Trimble CL Kosary C Trimble EL Long-term survival and patterns of care in woman with ovarian tumors of low malignant potential Gynecol Oncol 2002 86 34 37 10.1006/gyno.2002.6711 12079297

[B4] Fung MF Bryson P Johnston M Charmbers A Screening postmenopausal women for ovarian cancer: a systematic review J Obstet Gynaecol Can 2004 26 717 728 15307976 10.1016/s1701-2163(16)30643-0

[B5] Armstrong DK Bundy B Wenzel L Huang HQ Baergen R Lele S Copeland LJ Walker JL Bunger RA Intraperitoneal cisplatin and paclitaxel in ovarian cancer N Engl J Med 2006 354 34 43 10.1056/NEJMoa052985 16394300

[B6] Alberts DS Liu PY Hannigan EV O'Toole R Williams SD Young JA Franklin EW Clarke-Pearson DL Malviya VK DuBeshter B Intraperitoneal cisplatin plus intravenous cyclophosphamide versus intravenous cisplatin plus intravenous cyclophosphamide for stage III ovarian cancer N Engl J Med 1996 335 1950 1955 10.1056/NEJM199612263352603 8960474

[B7] Markman M Bundy BN Alberts DS Fowler JM Clark-Pearson DL Carson LF Wadler S Sickel J Phase III trial of standard-dose intravenous cisplatin plus paclitaxel versus moderately high-dose carboplatin followed by intravenous paclitaxel and intraperitoneal cisplatin in small-volume stage III ovarian carcinoma: an intergroup study of the Gynecologic Oncology Group, Southwestern Oncology Group, and Eastern Cooperative Oncology Group J Clin Oncol 2001 19 1001 1007 11181662 10.1200/JCO.2001.19.4.1001

[B8] Wenzel LB Huang HQ Armstrong DK Walker JL Cella D Health-related quality of life during and after intraperitoneal versus intravenous chemotherapy for optimally debulked ovarian cancer: a gynecologic oncology group study J Clin Oncol 2007 25 437 443 10.1200/JCO.2006.07.3494 17264340

[B9] Piccart MJ Bertelsen K James K Cassidy J Manginol C Simonsen E Stuart G Kaye S Vergote I Blom R Grimshaw R Atkinson RJ Swenerton KD Trope C Nardi M Kaern J Tumolo S Timmers P Roy JA Lhoas F Lindvall B Bacon M Birt A Andersen JE Zee B Paul J Baron B Pecorelli S Randomized intergroup trial of cisplatin-paclitaxel versus cisplatin-cyclophosphamide in women with advanced epithelial ovarian cancer: three-year results J Natl Cancer Inst 2000 92 699 708 10.1093/jnci/92.9.699 10793106

[B10] Kajiyama H Shibata K Terauchi M Ino K Nawa A Kikkawa F Involvement of SDF-1α/CXCR4 axis in the enhanced peritoneal metastasis of epithelial ovarian carcinoma Int J Cancer 2008 122 91 99 10.1002/ijc.23083 17893878

[B11] Yagi H Yotsumoto F Miyamoto S Heparin-binding epidermal growth factor-like growth factor promotes transcoelomic metastasis in ovarian cancer through epithelial-mesenchymal transition Mol Cancer Ther 2008 7 3441 3451 10.1158/1535-7163.MCT-08-0417 18852147

[B12] Slack-Davis KK Atkins KA Harrer C Hershey ED Conaway M Vascular cell adhesion molecule-1 is a regulator of ovarian cancer peritoneal metastasis Cancer Res 2009 69 1469 1476 10.1158/0008-5472.CAN-08-2678 19208843

[B13] Pickl M Ries CH Comparison of 3D and 2D tumor models reveals enhanced HER2 activation in 3D associated with an increased response to trastuzumab Oncogene 2009 28 461 468 10.1038/onc.2008.394 18978815

[B14] Laurent-Puig P Lievre A Blons H Mutations and response to epidermal growth factor receptor inhibitors Clin Cancer Res 2009 15 1133 1139 10.1158/1078-0432.CCR-08-0905 19228718

[B15] Cabanes A Briggs KE Gokhale PC Treat JA Rahman A Comparative in vivo studies with paclitaxel and liposome-encapsulated paclitaxel Int J Oncol 1998 12 1035 1040 9538125 10.3892/ijo.12.5.1035

[B16] Batist G Ramakrishnan G Rao CS Chandrasekharan A Gutheil J Guthrie T Shah P Khojasteh A Nair MK Hoelzer K Tkaczuk K Park YC Lee LW Reduced cardiotoxicity and preserved antitumor efficacy of liposome-encapsulated doxorubicin and cyclophosphamide compared with conventional doxorubicin and cyclophosphamide in a randomized, multicenter trial of metastatic breast cancer J Clin Oncol 2001 19 3439 3441 10.1200/JCO.2001.19.5.1444 11230490

[B17] Allen TM Austin GA Chonn A Lin L Lee KC Uptake of liposomes by cultured mouse bone marrow macrophages: influence of liposome composition and size Biochim Biophys Acta 1991 1061 56 64 10.1016/0005-2736(91)90268-D 1995057

[B18] Shimada Y Yoshino M Wakui A Nakao I Futatsuki K Sakata Y Kambe M Taguchi T Ogawa O Phase II study of CPT-11, a new Camptothecin derivative, in metastatic colorectal cancer. CPT-11 gastrointestinal cancer study group J Clin Oncol 1993 11 909 913 8487053 10.1200/JCO.1993.11.5.909

[B19] Masuda N Fukuoka M Kusunoki Y Matsui K Takifiji N Kudoh S Negoro S Nishioka M Nakagawa K Takada M A new derivative of Camptothecin for the treatment of refractory or relapsed small-cell lung cancer J Clin Oncol 1992 10 1225 1229 1321891 10.1200/JCO.1992.10.8.1225

[B20] Matsuzaki T Takagi A Furuta T Ueno S Kurita A Nohara G Kodaira H Sawada S Hashimoto S Antitumor activity of IHL-305, a novel pegylated liposome containing irinotecan, in human xenograft models Oncol Rep 2012 27 189 197 21935577 10.3892/or.2011.1465

[B21] Shmeeda H Tzemach D Mak L Gabizon A Her2-targeted pegylated liposomal doxorubicin: Retention of targetspecific binding and cytotoxicity after in vivo passage J Control Release 2009 136 155 160 10.1016/j.jconrel.2009.02.002 19331844

[B22] Lin Y-Y Chang C-H Lu Y-C Hwang J-J Tseng Y-L Lin W-J Ting G Wang H-E Evaluation of pharmacokinetics of 111In-labeled VNP-PEGylated liposome after intraperitoneal and intravenous administration in a tumor/ascites mouse model Cancer Biother Radiopharm 2009 24 453 460 10.1089/cbr.2008.0572 19694580

[B23] Liau S-S Jazag A Ito K Whang EE Overexpression of HMGA1 promotes anoikis resistance and constitutive Akt activation in pancreatic adenocarcinoma cells British J Cancer 2007 96 993 1000 10.1038/sj.bjc.6603654 PMC2360112 17342093

[B24] McGuire WP Hoskins WJ Brady MF Kugera PR Partridge EE Look KY Clarke-Pearson DL Davidson M Cyclophosphamide and cisplatin compared with paclitaxel and cisplatin in patients with stage III and stage IV ovarian cancer N Engl J Med 1996 334 1 6 10.1056/NEJM199601043340101 7494563

[B25] Takagi A Watanabe M Ishii Y Morita J Hirokawa Y Matsuzaki T Shiraishi T Three-dimensional cellular spheroid formation provides human prostate tumor cell with tissue-like features Anticancer Res 2007 27 45 54 17352215

[B26] Gabizon A Tzemach D Gorin J Mak L Amitay Y Shmeeda H Zalipsky S Improved therapeutic activity of folate-targeted liposomal doxorubicin in folate receptor-expressing tumor models Cancer Chemother Pharmacol 2009 66 43 52 19779718 10.1007/s00280-009-1132-4

[B27] Estep AL Palmer C McComick F Rauen KA Mutation analysis of BRAF, MEK1 and MEK2 in 15 ovarian cancer cell lines: implications for therapy PLoS One 2007 2 e1279 10.1371/journal.pone.0001279 18060073 PMC2093994

[B28] Sako A Kitayama J Koyama H Ueno H Uchida K Hamada H Nagawa H Transduction of soluble Flt-1 gene to peritoneal mesothelial cells can effectively suppress peritoneal metastasis of gastric cancer Cancer Res 2004 64 3624 3628 10.1158/0008-5472.CAN-04-0304 15150121

[B29] Sugiyama T Kamura T Kigawa J Terakawa N Kikuchi Y Kita T Suzuki M Sato I Taguchi K Clinical characteristics of clear cell carcinoma of the ovary Cancer 2000 88 2584 9 10.1002/1097-0142(20000601)88:11<2584::AID-CNCR22>3.0.CO;2-5 10861437

[B30] Ballard BE Biopharmaceutical considerations in subcutaneous and intramuscular drug administration J Pharm Sci 1968 57 357 378 10.1002/jps.2600570301 4871917

